# Characteristics of asymptomatic *Plasmodium* spp. parasitaemia in Kwahu-Mpraeso, a malaria endemic mountainous district in Ghana, West Africa

**DOI:** 10.1186/s12936-015-1066-8

**Published:** 2016-01-22

**Authors:** Ewurama D. A. Owusu, Vincent Buabeng, Samuel Dadzie, Charles A. Brown, Martin P. Grobusch, Petra Mens

**Affiliations:** Department of Infectious Diseases, Division of Internal Medicine, Academic Medical Centre, Centre of Tropical Medicine and Travel Medicine, University of Amsterdam, Amsterdam, The Netherlands; Department of Medical Laboratory Sciences, School of Biomedical and Allied Health Sciences, College of Health Sciences, University of Ghana, Accra, Ghana; Medical Laboratory Department, Atibie Government Hospital, Kwahu-Atibie, Ghana; Medical Entomology Unit, Department of Parasitology, Noguchi Memorial Institute of Medical Research, University of Ghana, Accra, Ghana; Centre de Recherches Médicales de Lambaréné (CERMEL), Hôpital Albert Schweitzer, Lambaréné, Gabon; Institute of Tropical Medicine, University of Tübingen, Tübingen, Germany; Department of Parasitology, KIT Biomedical Research Institute, Amsterdam, The Netherlands

**Keywords:** Asymptomatic malaria, *Plasmodium* parasitaemia, Highland, Ghana, Sickle cell trait, *Anopheles gambiae*

## Abstract

**Background:**

Malaria control efforts in Ghana have reduced the countrywide average malaria prevalence from 71 % in 2000 to about 51 % in 2012; however, its main focus is on symptomatic malaria. If further progress is to be made, parasite reservoirs in asymptomatic carriers need to be moved into focus. This study profiles asymptomatic *Plasmodium* spp. parasitaemia amongst residents of mountainous Kwahu-Mpraeso in the Eastern region of Ghana.

**Methods:**

A cross-sectional study of 360 residents was carried out from October to December 2013. This included recording demographics, malaria testing of asymptomatic residents, and gathering of their malaria history. Assessment of malaria transmission was done with molecular identification of vectors, determination of sporozoite rate, insecticide resistance status and biting pattern. Univariate and multivariate analysis were used to establish risk determinants.

**Results:**

In Mpraeso, in the Kwahu highland of Eastern Region, children were at higher risk of asymptomatic parasitaemia, thereby contributing to the parasite reservoir and hence sustained malaria transmission. As well, findings suggested Hb AC genotype influenced susceptibility to asymptomatic malaria with 8.03-fold increase in odds (univariate) and 11.92-fold higher odds (multivariate) than the normal Hb AA. The mosquito vector predominant in the area was *Anopheles gambiae* sensu stricto of the homozygous pyrethroid resistant form (RR); with biting mainly occurring indoors.

**Conclusion:**

For an effective malaria control in this area, interventions should be formulated and implemented to target asymptomatic parasite reservoirs; especially in children and people with Hb AC. The dominant vector species *An. gambiae s.s*. and its feeding patterns of biting indoors should also be considered.

## Background

Ghana, a malaria endemic country, is actively fighting against this deadly disease which still killed about 2500 people out of 1,639,451 confirmed cases in 2013 [1]. The entire population of 25.9 million people is at risk of malaria [1, 2]; even though transmission is more intense in the rural areas, relative to the urban areas [2, 3]. The focus of malaria control in Ghana is on preventive measures such as use of long-lasting insecticidal nets (LLIN) and timely diagnosis and treatment in order to reduce the burden of disease [2, 4]. This has resulted in a noteworthy decline in national malaria prevalence over the years [2] from 71 % in 2000 [5] to about 51 % in 2012 [4].

It seems that a blanket approach of interventions by the Ministry of Health and the Ghana Malaria Control Programme, such as roll-out of LLIN, indoor residual spraying (IRS), change of first-line treatment to artemisinin-based combination therapy (ACT), shift from presumptive treatment to diagnostic + treatment and availability of WHO pre-qualified malaria rapid diagnostic test kits (RDT), has worked in Ghana to reduce malaria cases and deaths [4]. Currently, studies are focusing on elements that are essential in formulating tailor-made interventions for the different populations, such as parasite presence in humans and resistance to drugs [6–8], resistance of *Anopheles* vectors to insecticides [9–13], knowledge and health-seeking behaviour of the population affected [14–16], and the burden that malaria poses on specific risk groups like pregnant women [17–19]. However, a lot more needs to be done in these areas.

Next to symptomatic malaria patients that visit health clinics for treatment, asymptomatic carriers of malaria parasites need to be appropriately treated as well in order to reduce or even interrupt transmission. Studies have shown that in areas of intense malaria transmission, such as Ghana, asymptomatic children are major carriers of *Plasmodium falciparum* [20, 21] and, therefore, contribute to the parasite reservoir [22, 23]. Other patient groups that may need a more specific, rather than a one-size-fits-all, approach are individuals with haemoglobinopathies, such as sickle cell disease (SCD). The sickle cell trait (Hb AS) is known to grant a certain degree of protection against severe malaria whilst heterozygosity worsens the course of disease [24, 25].

Ghana is known to have up to 18.8 % of its population harbouring the HbS allele [26], with about 2 % of new-borns having SCD [27]. Haemoglobin C is also relatively common in West Africa [25, 28, 29], Ghana included [29]. In both Hb S and Hb C mutations occur in the 6th position in the beta-globin amino acid sequence (β-6) such that glutamic acid is substituted with lysine (Hb C) or valine (Hb S) [28]. Some work has been done on the influence of haemoglobin S and C in asymptomatic *Plasmodium* spp. carriers in the northern part of Ghana [20, 24], where an increased risk for *Plasmodium malariae* and *P. falciparum* infections was observed in people who were homozygous HbCC [20]. It was also shown that although Hb AS offered better protection against malaria, Hb AC reduced the risk of severe malaria [24]. In order to effectively control malaria in Ghana, there is a need to better understand the complexities of asymptomatic malaria, including its interaction with SCD.

Next to control in humans, many programmes also intervene at the vector level. However, this is being hampered by the emergence of resistant mosquitoes to the currently approved pyrethroid, used in indoor spraying and insecticide-treated nets [30]. In addition, mosquitoes are adapting their feeding behaviour in response to the use of preventive measures, such as LLIN [31, 32]. How these characteristics are expressed in the highlands of forest ecological zones in Ghana is unknown and needs to be assessed, if situation-specific, well-adapted malaria control policies are to be formulated.

In order to maintain this momentum of progress and to assess what interventions may help to reduce the malaria burden further; more information is needed on the above mentioned aspects. Therefore, this study set out to prospectively assess the asymptomatic malaria parasite burden in a rural population in the Kwahu highland of Eastern Region of Ghana, the influence of SCD, and some vector characteristics in this area.

## Methods

### Study sites and population

Kwahu-South is one of the districts of the Eastern region of Ghana, and Mpraeso is its capital. The district is 1876 sq km in size and located between latitude 06°35′36″N and longitude 00°44′05″W. Mpraeso is divided into six clusters: Ntuntuagya, Akropong, Plot su, Lion House, Nsuase and Kwasifori and has an approximate total population of 11,500 (Kassim Basit, personal communication). It lies in the tropical rain forest malaria-epidemiological zone [33] and has many streams and marshy areas. Kwahu, like other parts of the southern half of Ghana, has two main rainy seasons: major rainy season from May to June and minor rainy season from September to October. In general, malaria occurs all year round, as is the case everywhere in Ghana [11, 34]. It is the commercial hub of the rural mountain peak (788.21 m). For this reason only residents who had lived there for a minimum of 6 months were included in the study. Individuals of all ages were considered in this study, unless the required number for a particular age-group reached its limit.

### Study design and participant selection

This cross-sectional survey was done between October and December of 2013 to obtain baseline data of the malaria situation in the Mpraeso area. The study comprised of two parts: a questionnaire-laboratory survey and an entomological survey. Permission was sought from the District Health Directorate and household heads before commencement. Each cluster was stratified into age groups (0–5, 6–10, 11–15, 16–20, 21–40 and over 40 years) for which systematic selection from every 8th household was done. Sixty participants in each cluster were recruited. All the members of each household visited were encouraged to enrol in the study. Each data collector was rigorously trained in questionnaire administration and sample-taking. The questionnaires, which requested demographic information (e.g. sex, age and education), malaria prevention measures (ITN, coil and spray usage), exposure to mosquitoes (sleeping and waking times), recent use of anti-malarials, as well as fever management practices, were pre-tested prior to data collection. Residents who had malaria symptoms, e.g. fever, chills, headache, generalized body and joint pains, were excluded from this study. Also those who had recently been to the hospital with complaints suggestive of malaria were not included. Each participant was asked to first fill a questionnaire; and consecutively, blood was collected. For those who were illiterate, the trained sample collectors filled the questionnaire for them when they provided answers to the relevant questions. Parents/guardians of underage children provided responses on their behalf. For the entomological survey, two households in each cluster were selected and permission sought from the heads of households a few days before the survey.

### Ethics

Ethical clearance was granted by the Ghana Health Service Ethics Review Committee (GHS-ERC: 08/07/13) as well as the Noguchi Institutional Review Board (NMIMR-IRB CPN 067/13-14). Informed consent was obtained from willing participants before samples were collected.

### Laboratory survey

First Response^®^ Malaria Ag *P. falciparum* (HRP2) malaria rapid diagnostic test kit (RDT) was used to test finger prick capillary blood of each participant for presence of *P. falciparum*. Residents who tested positive were given artemisinin-based combination therapy (ACT), which is the standard malaria treatment in Ghana. Both thick and thin blood smears were prepared for microscopy examination to detect and identify parasites, respectively. The blood smears were air-dried and sent to the Medical Laboratory Department of the Atibie Government Hospital where thin blood smears were fixed with 100 % methanol. Both thick and thin films were stained with 10 % Giemsa and examined by two expert microscopists in the laboratory. If there were no asexual parasites observed in 200 high powered visual fields, a sample was recorded as negative; samples were considered positive if any amount of asexual parasites was observed per 200 white blood cells. The expertise of a third microscopist was sought when results were inconclusive. A few drops of blood (about 1 ml) were collected for Hb genotyping [35]. Blood was categorized into HbAA (normal), HbAS (trait), HbAC (trait), HbSS (SCD) or HbSC (SCD).

### Entomological study

The human landing catch method was used to catch mosquitoes each night for a total of 24 man nights, from two selected households at the beginning and end of each cluster area. This study was done in November–December, which is just after the September–October minor rainy season. The study area is at an elevation of 788.21 m. All recruited team members (except supervisors who did the training) were thoroughly trained local residents who were acclimatized to the parasite species of that area. They were also given prophylaxis prior to and during the study period. Two teams of four catchers each, evenly distributed indoors and outdoors, caught mosquitoes attempting to bite them; and kept them in paper cups covered by mesh. Collectors worked from 6 pm to 6am, rotating between indoors and outdoors every hour to reduce bias of individual attractiveness or repulsion to mosquitoes.

### Mosquito processing

After collection mosquitoes were knocked out in the freezer for a few minutes, sorted and morphologically identified (sex and species identification) using taxonomic keys [36] in the morning, immediately upon return from the field. Female anophelines were stored in Eppendorf tubes with desiccants and transported to the laboratory of the Parasitology Department of Noguchi Memorial Institute for Polymerase Chain Reaction (PCR) and Enzyme-Linked Immunosorbent Assay (ELISA). Genomic DNA was extracted from the legs of individual female anophelines for the identification of the sibling species of the *Anopheles gambiae* complex by PCR [37]. For the identification of the Savannah (S) form and Mopti (M) molecular forms of *Anopheles gambiae* sensu stricto, *Hhal* restriction of the PCR product obtained was carried out according to the method of Fanello et al. [38]. The presence of the West African mutation (leucine to phenylalanine L1014P) pyrethroid *knock*-*down resistance* gene (*kdr*) was also detected using PCR [39]. These were classified as homozygous resistant (RR), heterozygous resistant/susceptible (RS) and homozygous susceptible (SS). The presence of circumsporozoite antigens (CSA) of *P. falciparum, Plasmodium ovale* and *P. malariae* in the salivary glands of anopheline mosquitoes was determined by processing the thorax and head, and using ELISA [40].

### Statistical analysis

Data obtained from both laboratory and questionnaires were entered into Microsoft Excel and statistically analysed using IBM SPSS 20. Both univariate and multivariate logistic regression were used to determine the effect of the independent variable on the primary outcome of malaria positivity, individually and as a group, respectively. A *p* value of <0.05 was considered statistically significant. The number of *Anopheles* biting per person per night was the calculation used for the human biting rate, and the proportion of anophelines that tested positive for *P. falciparum* circumsporozoite antigen (*Pf*CSP) was used to calculate the sporozoite rate. A product of sporozoite rate and human biting rate at a given place and time interval yielded the entomological inoculation rates.

## Results

### Malaria status and Hb genotype of residents

Out of the total 360 residents that took part in this study, 43 (11.94 %) were parasitaemic for *Plasmodium* spp. (microscopy), not exhibiting any malaria symptoms. Six residents had both *P. falciparum* and *P. malariae* parasites, whilst three had only *P. malariae* infection.

There was an equal distribution of participants amongst the different age groups (60 each) of <5, 6–10, 11–15, 16–20, 21–40 and >40 years; as well as an equal distribution across the 6 different clusters (60 each). The independent parameters of malaria positive residents are summarised in Table 1. The highest number of malaria positives was observed in residents of Lion House (9; 20.9 %), with Ntuntuagya recording the lowest (5; 11.6 %). Children aged 11–15 years were the most infected (11; 25.6 %), with adults over 20 years being the least infected (5; 11.6 %) for each age group. The male:female ratio of those who tested positive in this study was 21:22, with the widest disproportion occurring in Plot su where only one female had asymptomatic parasitaemia as compared to seven males. About 32 % of those with malaria parasites did not have any form of formal education, and less than 70 % could recall their last malaria episode. There was no use of IRS in the study area and only a quarter of those who had malaria (25.6 %) used LLIN. Many of them (32.7 %) did not use any form of protection against mosquito bites. When asked why people abstained from using mosquito nets, most people (42 %) seemed to have more confidence in the use of mosquito coils and sprays. In the event of fever (which in local parlance is a synonym for malaria symptoms), only 4.7 % resorted to herbal medicine only. Many people either combined herbal with over-the-counter drugs (OTC) (34.9 %) or depended mainly on OTCs (25.6 %).

**Table 1:** Baseline data of residents who tested malaria positive in the six cluster areas of Mpraeso

	Malaria positive	Cluster						Total
		Ak	Kw	Lh	Ns	Nt	Pl	
*Plasmodium* spp.	*P. falciparum*							40 (93.0 %)
	*P. malariae*							3 (7.0 %)
	Mixed							6 (14.0 %)
Age band	<5	1 (2.3 %)	2 (4.7 %)	2 (4.7 %)	1 (4.7)	0 (0 %)	1 (2.3 %)	7 (16.3 %)
	6–10	2 (4.7 %)	1 (2.3 %)	2 (4.7 %)	2 (4.7 %)	1 (2.3 %)	1 (2.3 %)	9 (20.9 %)
	11–15	1 (2.3 %)	1 (2.3 %)	4 (9.3 %)	2 (4.7 %)	1 (2.3 %)	2 (4.7 %)	11 (25.6 %)
	16–20	1 (2.3 %)	0 (0 %)	0 (0 %)	1 (2.3 %)	1 (2.3 %)	3 (7 %)	6 (14 %)
	21–40	1 (2.3 %)	2 (4.7 %)	0 (0 %)	0 (0 %)	1 (2.3 %)	1 (2.3 %)	5 (11.6 %)
	>40	0 (0 %)	1 (2.3 %)	1 (2.3 %)	2 (4.7 %)	1 (2.3 %)	0 (0 %)	5 (11.6 %)
	Total	6 (14 %)	7 (16.3 %)	9 (20.9 %)	8 (18.6 %)	5 (11.6 %)	8 (18.6 %)	43 (100 %)
Sex	F	3 (7 %)	4 (9.3 %)	6 (14 %)	6 (14 %)	2 (4.7 %)	1 (2.3 %)	22 (51.2 %)
	M	3 (7 %)	3 (7 %)	3 (7 %)	2 (4.7 %)	3 (7 %)	7 (16.3 %)	21 (48.8 %)
	Total	6 (14 %)	7 (16.3 %)	9 (20.9 %)	8 (18.6 %)	5 (11.6 %)	8 (18.6 %)	43 (100 %)
Education	Nil	3 (7 %)	4 (9.3 %)	4 (9.3 %)	2 (4.7 %)	1 (2.3 %)	2 (4.7 %)	16 (32.2 %)
	Primary	2 (4.7 %)	2 (4.7 %)	1 (2.3 %)	4 (9.3 %)	2 (4.7 %)	3 (7 %)	14 (32.6 %)
	Secondary	0 (0 %)	1 (2.3 %)	4 (9.3 %)	2 (4.7 %)	2 (4.7 %)	3 (7 %)	12 (27.9 %)
	Tertiary	1 (2.3 %)	0 (0 %)	0 (0 %)	0 (0 %)	0 (0 %)	0 (0 %)	1 (2.3 %)
	Total	6 (14 %)	7 (16.3 %)	9 (20.9 %)	8 (18.6 %)	5 (11.6 %)	8 (18.6 %)	43 (100 %)
Last malaria episode	<3 months	0 (0 %)	2 (4.7 %)	1 (2.3 %)	3 (7 %)	0 (0 %)	1 (2.3 %)	7 (16.3 %)
	4–6 months	0 (0 %)	1 (2.3 %)	0 (0 %)	1 (2.3 %)	0 (0 %)	0 (0 %)	2 (4.7 %)
	7–12 months	1 (2.3 %)	0 (0 %)	0 (0 %)	1 (2.3 %)	0 (0 %)	0 (0 %)	2 (4.7 %)
	>1 year	1 (2.3 %)	0 (0 %)	0 (0 %)	2 (4.7 %)	0 (0 %)	0 (0 %)	3 (7 %)
	Can not remember	4 (9.3 %)	4 (9.3 %)	7 (16.3 %)	1 (2.3 %)	5 (11.6 %)	6 (14 %)	27 (62.8 %)
	Never had	0 (0 %)	0 (0 %)	1 (2.3 %)	0 (0 %)	0 (0 %)	1 (2.3 %)	2 (4.7 %)
	Total	6 (14 %)	7 (16.3 %)	9 (20.9 %)	8 (18.6 %)	5 (11.6 %)	8 (18.6 %)	43 (100 %)
Protection	LLIN	1 (2.3 %)	2 (4.7 %)	3 (7 %)	1 (2.3 %)	1 (2.3 %)	3 (7 %)	11 (25.6 %)
	IRS	0 (0 %)	0 (0 %)	0 (0 %)	0 (0 %)	0 (0 %)	0 (0 %)	0 (0 %)
	Others	4 (9.3 %)	3 (7 %)	3 (7 %)	4 (9.3 %)	2 (4.7 %)	2 (4.7 %)	18 (42 %)
	No protection	1 (2.3 %)	2 (4.7 %)	3 (7 %)	3 (7 %)	2 (4.7 %)	3 (7 %)	14 (32.7 %)
	Total	6 (14 %)	7 (16.3 %)	9 (20.9)	8 (18.6 %)	5 (11.6 %)	8 (18.6 %)	43 (100 %)
Approach to fever	Hospital	1 (2.3 %)	2 (4.7 %)	1 (2.3 %)	1 (2.3 %)	1 (2.3 %)	0 (0 %)	6 (14 %)
	Herbal	1 (2.3 %)	0 (0 %)	0 (0 %)	0 (0 %)	0 (0 %)	1 (2.3 %)	2 (4.7 %)
	OTC	1 (2.3 %)	3 (7 %)	3 (7 %)	3 (7 %)	0 (0 %)	1 (2.3 %)	11 (25.6 %)
	Combine	2 (4.7 %)	2 (4.7 %)	5 (11.6 %)	3 (7 %)	1 (2.3 %)	2 (4.7 %)	15 (34.9 %)
	No action	1 (2.3 %	0 (0 %)	0 (0 %)	1 (2.3 %)	3 (7 %)	4 (9.3 %)	9 (20.9 %)
	Total	6 (14 %)	7 (16.3 %)	9 (20.9 %)	8 (18.6 %)	5 (11.6 %)	8 (18.6 %)	43 (100 %)
SCD	AA	4 (9.3 %)	4 (9.3 %)	4 (9.3 %)	6 (14 %)	2 (4.7 %)	4 (9.3 %)	24 (55.8 %)
	AC	1 (2.3 %)	3 (7 %)	3 (7 %)	2 (4.7 %)	2 (4.7 %)	2 (4.7 %)	13 (30.2 %)
	AS	1 (2.3 %)	0 (0 %)	2 (4.7 %)	0 (0 %)	1 (2.3 %)	2 (4.7 %)	6 (14 %)
	Total	6 (14 %)	7 (16.3 %)	9 (20.9 %)	8 (18.6 %)	5 (11.6 %)	8 (18.6 %)	43 (100 %)

*Ak* Akropong, *Kw* Kwasifori, *Lh* Lion House, *Ns* Nsuase, *Nt* Ntuntuagya, *Pl* Plot su

A little under half of those with malaria had either haemoglobin AC (30.2 %) or AS (14 %). No resident with sickle cell disease (Hb SS/Hb SC) had asymptomatic malaria, even though 1.6 and 1.9 % of the population sampled carried Hb SS or Hb SC, respectively. Table 2 shows the univariate and multivariate regression analysis for predictor variables on malaria positivity. None of the variables studied except haemoglobin AC (both univariate and multivariate) and use of over-the-counter drugs (OTC) (multivariate) showed a significant risk outcome. In the univariate regression analysis, Hb AC had an eight-fold increase in odds, whilst when all predictor variables were taken into consideration in a multivariate regression analysis, it yielded an 11-fold increase in odds. However, there was no significant increase in odds when those who did not use any form of fever remedy were analysed alone, whereas there was a 2.86-fold increase during the multivariate regression analysis.

**Table 2:** Univariate and multivariate regression analysis of risk factors for asymptomatic malaria

	n (%)	Univariate analysis	Multivariate analysis
		odds ratio (CI), P value	odds ratio (CI), P value
Malaria positive	43 (11.9)	–	–
Age group			
<5	60 (16.67)	1	1
6–10	60 (16.67)	1.336 (0.46–3.86), 0.59	1.6 (0.26–9.70), 0.61
11–15	60 (16.67)	1.7 (0.61–4.73), 0.31	2.10 (0.31–14.17), 0.45
16–20	60 (16.67)	0.841 (0.27–2.67), 0.77	0.64 (0.09–4.41), 0.65
21–40	60 (16.67)	0.688 (0.21–2.3), 0.55	0.50 (0.1–2.51), 0.40
Over 40	60 (16.67)	0.688 (0.21–2.3), 0.55	0.82 (0.19–3.53), 0.79
Sex			
Female	196 (54.40)	1	1
Male	164 (45.56)	0.86 (0.46–1.6), 0.65	1.06 (0.49–2.27), 0.89
Cluster			
Ak	60 (16.67)	1	1
Kw	60 (16.67)	1.19 (0.38–3.78), 0.77	0.63 (0.15–2.59), 0.52
Lh	60 (16.67)	1.59 (0.53–4.78), 0.41	1.38 (0.33–5.74), 0.66
Ns	60 (16.67)	1.39 (0.45–4.27), 0.58	0.69 (0.18–2.72), 0.60
Nt	60 (16.67)	0.82 (0.24–2.84), 0.75	0.58 (0.13–2.58), 0.48
Pl	60 (16.67 %)	1.39 (0.45–4.27), 0.58	1.67 (0.43–6.41), 0.46
Protection			
A	23 (6.38)	1	1
Mc	48 (13.3)	1.1 (0.3–4.02), 0.89	1.05 (0.23–4.89), 0.95
Mn	74 (20.56)	0.34 (0.08–1.40), 0.14	0.28 (0.05–1.47), 0.13
Mns	30 (8.33)	0.34 (0.06–2.04), 0.24	0.22 (0.03–1.93), 0.17
Ms	48 (13.33)	0.55 (0.13–2.29), 0.41	0.47 (0.09–2.50), 0.37
Msc	18 (5.00)	1.36 (0.29–6.38), 0.70	1.65 (0.27–10.28), 0.59
N	108 (30.00)	0.71 (0.21–2.39), 0.58	0.55 (0.13–2.38), 0.42
SCD^a^			
AA	276 (76.67)	1	1
AC	30 (8.33)	8.03 (3.4–18.5), <0.05	11.92 (4.30–33.08), <0.05
AS	43 (11.94)	1.70 (0.65–4.44), 0.28	2.124 (0.69–6.54), 0.19
Last malaria episode			
<3 months	48 (13.33)	1	1
4–6 months	11 (3.06)	1.3 (0.23–7.33), 0.77	2.41 (0.32–17.88), 0.39
7–12 months	17 (4.72)	0.78 (0.15–4.18), 0.77	0.93 (0.133–6.60), 0.95
>1 year	15 (4.17)	1.47 (0.33–6.54), 0.62	1.41 (0.23–8.72), 0.71
Can not remember	251 (69.72)	0.70 (0.29–1.72), 0.45	0.69 (0.23–2.09), 0.51
Never had	18 (5.00)	0.73 (0.14–3.90), 0.72	0.61 (0.08–4.95), 0.64
Fever remedy			
Combine	152 (42.2)	1	1
Hospital	73 (20.23)	0.76 (0.29–2.03), 0.59	1 (0.32–3.13), 1
Herbal	23 (6.39)	0.81 (0.17–3.78), 0.79	1.40 (0.25–7.79), 0.7
Ignore	12 (3.33)	0.78 (0.09–6.38), 0.81	0.76 (0.06–10.05), 0.84
No remedy	37 (10.28)	1.98 (0.75–5.24), 0.17	2.88 (0.85–9.73), 0.04
OTC	63 (17.5)	1.80 (0.78–4.13), 0.17	2.86 (1.05–7.80), 0.09
Education			
Nil	162 (45)	1	1
Primary	116 (32.22)	1.25 (0.59–2.68), 0.56	0.59 (0.14–2.49), 0.47
Secondary	74 (20.56)	1.77 (0.79–3.95), 0.17	1.97 (0.45–8.54), 0.37
Tertiary	8 (2.22)	1.30 (0.15–11.28), 0.81	2.26 (0.19–26.85), 0.52

Protection here is defined as: *A* all of the below, *Mc* mosquito coil, *Mn* mosquito net (LLIN), *Mns* mosquito net (LLIN) and spray, *Ms* mosquito spray, *Msc* mosquito spray and coil, *N* no protection

^a^The frequency of Hb SC and Hb SS for entire population studied were 6 (1.7 %) and 5 (1.4 %) respectively

### Entomology

**Fig. 1:**
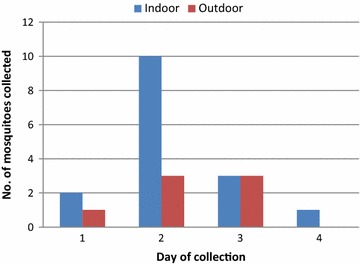
Daily capture of mosquitoes using HLC in the Mpraeso area

**Fig. 2:**
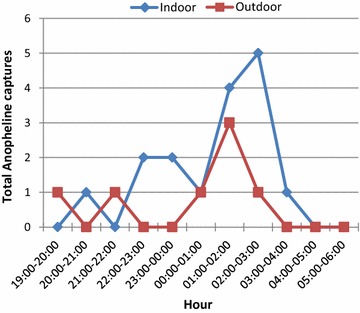
Hourly biting pattern of Anophelines using HLC in Mpraeso

A total of 23 mosquitoes were captured in the 24 collecting nights (Fig. 1). Majority of the mosquitoes collected (91.35 %) were *Anopheles gambiae s.s.* while *Anophelespharoensis* and *Anopheles funestus* made up 4.3 %. Indoor biting collection comprised 69.6 % of the samples and 30.4 % were from outdoor biting collection. Indoor biting gradually increased from 20:00 to 21:00 h, peaked at 2–3 h and was sustained till dawn. Outdoor biting started rising from 19 to 20 h and peaked at 1–2 h, but declined at 2–3 h (Fig. 2).

**Fig. 3:**
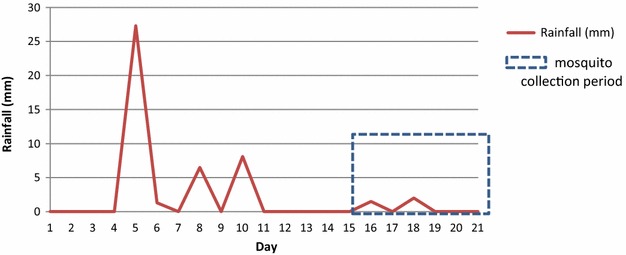
Mean daily rainfall within the 2 weeks of data collection

The study area at an elevation of 788.21 m had maximum 2.0 mm and minimum 1.5 mm rainfall during the mosquito collection, but rainfall was as high as 27.3 mm in the preceding week (Fig. 3).

**Fig. 4:**
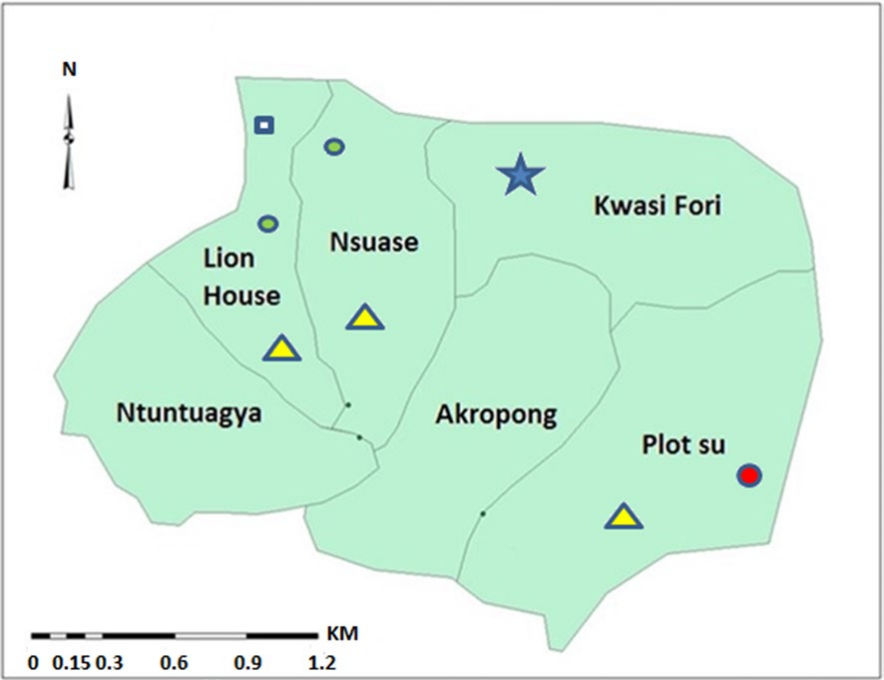
The six clusters in Mpraeso study area indicating outstanding *Plasmodium* spp. carriage and entomological data. *Blue star* highest *Anopheles gambiae* population. *Red circle* the only M form and heterozygous kdr RS identified. *Yellow triangle* clusters with the highest *Plasmodium* spp. carriage during the study. *Green circle* clusters with over half the population studied having a history of malaria in less than 3 months. *Blue square* area with at least half the population studied agreeing to the use of LLIN

All of the *An. gambiaes.s.* were of the S form; except one which was an M form (*Anopheles coluzzii*) (Fig. 4). For two samples, there was no amplification of the *kdr* gene. All the rest (95.2 %) were homozygous resistant (RR); with the exception of 1 (4.8 %), which was heterozygous resistant (RS) (Fig. 4). The sporozoite rate of *An. gambiaes.s.* was 4.3 % (1/23), whilst the human biting rate was 0.96 bites/man/night. EIR was thus calculated to be 0.041 infective bites/man/night, which translated to 15 infective bites/man/year.

## Discussion

Malaria parasite prevalence in asymptomatic individuals was 11.94 %; despite the fact that only limited amounts of mosquitoes could be observed in the area. When analysed together with all the predictor values or alone, residents with Hb AC and those who did not use any form of fever remedy were at a significantly higher risk of malaria. All other factors studied did not significantly influence *Plasmodium* spp. carriage in this area.

Of the six clusters studied, Lion House had the highest asymptomatic malaria carriers whilst Ntuntuagya recorded the lowest. However, both univariate and multivariate did not show a significant increase or decrease in the odds of malaria infection risk with respect to residential clusters. As expected and reported before [41], lack of LLIN usage and delayed treatment resulted in increased prevalence.

Many residents used mosquito coils that are readily available on the local markets; a similar pattern is observed in other areas of Ghana. In a previous study [41], as many as 43 % of the households in Ghana across the entire socio-economic spectrum used mosquito coils every day as a substitute for mosquito nets, even when they owned LLIN. That study also showed that people used LLIN more if they bought them themselves instead of the freely distributed ones. This could explain why there was low use of LLIN by participants in this study in spite of recent free LLIN distribution in the area.

The only significant finding of a possible increased risk of asymptomatic malaria by all counts was with Hb AC. Hb AC has previously been associated with reduced odds of severe malaria [24], but very little data exists on the effect of Hb AC on asymptomatic malaria. One of the few studies done was in Northern Ghana [20], which showed there was no significant effect of Hb AC on asymptomatic malaria, even though the distribution of hemoglobinopathies across the population was similar in both studies.

Children, especially those between 11 and 15 years, had a higher rate of asymptomatic parasitaemia as compared to adults. This is in agreement with other studies in Ghana that showed older children to be the major asymptomatic carriers of *P. falciparum* [20, 21]. Reasons for this are not explored further in this study, but the finding does highlight that special attention needs to be given to them with respect to prevention of malaria and their role in transmission of the disease.

The dominant vector in Kwahu-Mpraeso was the Savannah (S) form of *An. gambiae s.s.* This is in line with other studies that have collected data in similar environmental conditions just after the rains. The abundance of the S form in this area is, therefore, congruent with literature which shows that it relies on rainfall to breed, and that it ebbs during the dry season [42–44]. It is also influenced by elevation and temperature [44]. However, the low numbers of mosquitoes collected may be due to the long rainy days encountered prior to collection, which could have washed away the breeding sites of the malaria vectors. Mosquito biting started quite early in the evening and was sustained till dawn; a pattern also observed in Northern Ghana [43]. The majority of the biting took place indoors. Nearly all mosquitoes captured carried the West African *kdr* mutation (*kdr*-*w*), the majority of which were homozygous resistant (RR) for the knock-down resistance gene. This is similar to another study which detected higher allelic R frequency in Ghana [10]. The only RS heterozygous mosquito was the only M form and it was identified in the Plot su cluster. In a study where mosquitoes were exposed to pyrethroid (used in ITNs), none of the dead mosquitoes had RR genotype whilst 70 % of survivors carried the *kdr* mutation [30]. The *kdr*-*w* mutation has been shown to be associated with resistant phenotypes to pyrethroids [30], which is the insecticide type used in LLIN. In this area, 38.1 % of residents use LLIN and this might have an effect on the level of resistance to pyrethroids [10]. However, since this study did not include detection of resistant phenotypes, there is a need for further research on resistance in this study area, which should include other collection methods and collection spanning the different seasons.

Certain limitations were encountered during the implementation and analysis of this study. This was a cross-sectional study, as such subsequent increase in study duration and sample size could unravel possible associations between a number of predictor values and malaria positivity, as well as the true depiction of the mosquito vectors in the area.

As with every cross sectional studies, causal inferences might also not be appropriately deduced. Repeated measures at least after each of the rainy seasons as well as the dry season may be needed for some of the predictors in this study. For instance, data collection of mosquitoes was in November–December, just at the end of the minor rainy season in Ghana. There were also six continuous rainy days just prior to and during the mosquito collection, which could have washed away temporary breeding sites. The different forms usually exhibit strong association with particular regional habitats which are subject to seasonal variations, particularly in rainfall [42, 45]. Despite these limitations, this study provides novel data on the sickle cell status and their relation with asymptomatic carriage.

This study shows that in the highlands of Ghana, indeed, a high proportion of individuals may carry malaria parasites without exhibiting symptoms, and are, therefore, important reservoirs for malaria transmission in the area. If one wants to interrupt the transmission further, attention needs to be given to proper use of protective measures especially for young adolescents. This group may benefit from tailor-made education campaigns specifically adapted to their own interests, which may be different from those of the adult population. In addition, emphasis needs to be made on proper use of LLINs as they are proven to prevent mosquito bites during sleeping time, which is the time at which exposure mostly occurs in this area. Although the majority of mosquitoes found in this area may be resistant to pyrethroids used in bed nets, the physical barrier may still be effective in reducing part of the mosquito bites. The presence of high level *kdr*-*w* mutants does however flag up the need to look for alternative ways of protection to be included in the malaria control program as well. Furthermore, control programmes would benefit from further studies towards the relation of Hb AC individuals as they have shown to be at higher risk in all age groups for asymptomatic parasite carriage compared to other Hb types. Focused preventive measures, preventive treatment or more frequent screening could help in reducing the burden of malaria in this group and thereby the overall burden of the communities.

## Conclusions

This study has shown that the asymptomatic carrier rate in Mpraeso in the Kwahu highland of Eastern Region of Ghana is high. It suggested a possible influence of Hb AC genotype and provided an overview of the mosquito vectors prevalent with their feeding patterns. These individual factors need to be considered in order to make malaria control efforts more effective.
